# The psychometric properties of the self-expression and emotion regulation in art making scale (SERAMS) in Chinese university students majoring in art and design

**DOI:** 10.3389/fpsyg.2026.1586568

**Published:** 2026-07-15

**Authors:** Aifang Yu, Zhidao Shi

**Affiliations:** 1 Department of Public Art and Design, School of Fine Arts, Hangzhou Normal University, Hangzhou, China; 2 Interdisciplinary Research Center for Digital Intelligence Design, Fine Arts School, Hangzhou Normal University, Hangzhou, China; 3 Clinical Research Center for Mental Disorders, Shanghai Pudong New Area Mental Health Center, School of Medicine, Tongji University, Shanghai, China

**Keywords:** art making, emotion regulation, reliability, scale revision, self-expression, university students, validity

## Abstract

**Aim:**

Translating the Self-Expression and Emotion Regulation in Art Therapy Scale (SERATS) into Chinese and adapting it for use by Chinese university students majoring in art and design.

**Methods:**

After translating the SERATS into Chinese, we adapted it to the Self-Expression and Emotion Regulation in Art Making Scale (SERAMS). A total of 209 university students majoring in art and design were recruited to examine the reliability and validity of the scores derived from the SERAMS; of these participants, 121 were retested 2 weeks later. Another 200 university students majoring in art and design were selected to perform Confirmatory Factor Analysis (CFA).

**Results:**

The SERAMS is unidimensional and consists of nine items. The explained variance of SERAMS is 51.124%. CFA validated that the one-factor model fit with the data of SERAMS. Standardized factor loadings ranged between 0.688 and 0.733. The Cronbach’s alpha coefficient of SERAMS was 0.880. Correlation analysis was performed between SERAMS and The General Self-Efficacy Scale (GSES) to calculate the criterion-related validity (*r* = 0.571 *p* < 0.001). The Pearson correlation coefficient for SERAMS’ test–retest reliability was *r* = 0.889 (*p* < 0.001). SERAMS’s split-half reliability was 0.875 (Spearman-Brown coefficient).

**Conclusion:**

SERAMS has relatively good reliability and validity. It can be used to assess the self-expression and emotion regulation abilities of Chinese university students majoring in art and design.

## 1 Introduction

Self-expression and emotional regulation are two important elements of university art and design education. Through self-expression, art students convey their unique perspectives, identities, and emotions in the process of art making, transforming their rich inner worlds into tangible art forms (Al-Zadjali, 2024; Eisner, 2002; Richards, 2007a). This process is closely related to the highly regarded originality in art making (Csikszentmihalyi, 1997). Art students create works rich in personal meaning through active self-expression, which enhances their artistic design practice skills and cultivates their sense of ownership in the creative process (Runco, 2014), thereby increasing their sense of achievement and improving their professional competitiveness (Charest, 2020). Emotion regulation in art and design education refers to the ability of art students to effectively manage and deal with their emotions during the art making process. Effective emotion regulation enables art students to effectively cope with feelings of frustration, self-doubt, and uncertainty arising during the creative process while maintaining focus and resilience (Gross, 2015), navigating strong creative emotions such as anxiety or inspiration during the creative process, preventing mental chaos, and ensuring that the creative process is balanced and orderly, to hold a high-quality artistic output (Gao, 2023).

In art and design education, self-expression and emotional regulation capabilities help students cultivate critical thinking and emotional depth in their creative endeavors (Liu et al., 2024). They also enable students to integrate technical proficiency with emotional intelligence, laying the foundation for innovation-driven careers after graduation.

Chinese university students majoring in art and design often exhibit deficiencies in self-expression during their creative processes, with their works lacking originality and personal distinctiveness, favoring imitation of existing styles over bold innovation (Dou, 2015). These works not only reflect hesitation to break conventional patterns, leaning more toward reliance on replicated techniques rather than expressing personal viewpoints but also demonstrate a habitual dependence on safe, repetitive styles, making it difficult to showcase creative expression (Fung Shung-Yu and Choi Yuet-Ngor, 2001). A primary reason for this weakness in self-expression is the test-oriented model of China’s university entrance examinations for art majors, which prioritizes technical proficiency over creativity. This test-oriented education tends to restrict art students’ creative development, resulting in rigid thinking patterns. In order to successfully pass the university entrance exam for art majors, they can only focus on skill training, repeating and imitating, again and again, those successful examples who once got high scores in the entrance exam. In the long run, they do not dare to explore their inner voices and lack the courage and confidence to express themselves in their art creations (Hong, 2020). Some students’ personality traits, cultural norms emphasizing conformity over individuality, and limited opportunities to engage in interdisciplinary creative practices may also be contributing factors (Fielding and Chung, 1998; Zhao et al., 2020).

The emotional regulation ability of university students majoring in art and design in China also faces particular challenges. Some art students lack the ability to process emotions constructively during their art making process. The greater the creative pressure, the more unstable their emotions become. During their creative process, when the instructors or classmates express differing opinions about their work, or when their creative ideas are nearly exhausted to the point where work cannot continue, or as the submission deadline of their work approaches, they often feel frustrated, anxious, detached from reality, and they may even wholly abandon their work and leave it to fate (Fengyu et al., 2010; Shong, 2013; Zheng, 2009). Their deficiencies in emotional regulation may be due to their previous lack of training on how to manage stress in a highly competitive academic environment, the teachers’ neglect of emotional literacy development for art students during the teaching process, or the general social stigmatization of individuals who express vulnerable emotions, which places them in an awkward situation where they experience difficulties in emotional regulation during the artistic creation process while also feeling ashamed to admit it. Of course, this may also be partly related to their personality (Fengyu et al., 2010; Shong, 2013; Zheng, 2009).

Deficiencies in self-expression and emotional regulation among Chinese university art students can impede their artistic growth, diminish their confidence in professional settings, weaken their ability to create works that evoke emotional or cultural resonance, and potentially lead to career burnout, ultimately undermining their potential for careers in art and design (Hong, 2020; Lowenfeld, 1957; Richards, 2007b; Waters, 2024). It is particularly urgent to conduct scientific research on the challenges these art students face in self-expression and emotional regulation during the artistic creation process. However, China still lacks standardized tools to measure these constructs for art and design university students’ art making processes. This gap restricts the in-depth advancement of related research.

The Self-Expression and Emotion Regulation in Art Therapy Scale (SERATS), developed by Suzanne Haeyen, is an established research tool. Initially designed to assess therapeutic outcomes of art therapy, SERATS aims to measure how individuals express and regulate emotions through artistic processes, demonstrating high reliability and validity in clinical populations (Haeyen et al., 2018; Haeyen and Noorthoorn, 2021). Given the need to evaluate self-expression and emotional regulation in art education, this study aims to translate and adapt the scale into Chinese, targeting Chinese university students in art and design majors to examine its psychometric properties.

Whether in art making process of art therapy or in art making process of art education, it is necessary to express inner thoughts and process emotions through art, which is particularly relevant for art and design students during their learning phase (Malchiodi, 2011; Runco, 2014). Previous studies have shown that research scales used in clinical fields can be effectively applied to healthy populations after appropriate adaptation (Cooper et al., 1987; Krupp et al., 1989), indicating the feasibility of this approach in the current study.

## 2 Materials and methods

This study consists of two parts:

Stage I: Translating SERATS into Chinese and adapting SERATS’s Chinese version to the Self-Expression and Emotion Regulation in Art Making Scale (SERAMS).

Stage II: Evaluating the psychometric properties of the SERAMS.

The study was conducted following the Declaration of Helsinki and was approved by the Fine Art School of Hangzhou Normal University (approval number 2024003). It was conducted from February 2024 to June 2024 in the Fine Art School of Hangzhou Normal University in Hangzhou City of Zhejiang Province, China.

### 2.1 Participants

Undergraduate and graduate students majoring in art and design were included in this study. Subjects were excluded from the study if they met the following exclusion criteria:

- those who were suffering from severe physical or mental illness and unable to complete the study,
- who refused to participate in this study.

### 2.2 Instruments

#### 2.2.1 General information questionnaire

It included items on participants’ socio-demographic information, such as gender, age, and discipline.

#### 2.2.2 SERATS

Haeyen developed SERATS, a measure of self-expression and emotion regulation during art therapy activities (Haeyen et al., 2018). SERATS is a 9-item, one-dimensional scale. The response format is a five-point Likert scale, ranging from never true (1) to almost always true (5). Cronbach’s alpha for SERATS ranged from 0.90 (Haeyen and Noorthoorn, 2021) to 0.94 (Haeyen et al., 2018). The test–retest correlation was r = 0.96 (Haeyen et al., 2018). In this study, we will translate and adapt the SERATS to measure the self-expression and emotional regulation of Chinese university students majoring in art and design during their art making.

#### 2.2.3 The general self-efficacy scale (GSES)

GSES is a questionnaire developed by Schwarzer and Jerusalem (1995) based on Bandura’s self-efficacy theory that assesses an individual’s general sense of self-efficacy. Its Chinese version was revised by Zhang and Schwarzer (1995). GSES is a 9-item, one-dimensional scale. The response format is a four-point Likert scale, ranging from not at all true (1) to exactly true (4). The total score on the scale ranges from 10 to 40; the higher the score, the stronger the self-efficacy of the individual (Ma et al., 2022). Research in fields such as art and education has found a correlation between an individual’s sense of self-efficacy and self-expression, as well as between an individual’s sense of self-efficacy and emotion regulation (Alessandri et al., 2015; Kaimal and Ray, 2017; Khastari and Asgari, 2019; Luberto et al., 2014; Yusefi et al., 2024). Therefore, this study will use GSES to evaluate the criterion-related validity of SERAMS.

### 2.3 Translation and cross-cultural adaptation of the SERATS

We contacted the developers of SERATS via email and received permission to revise the scale. A cross-cultural adaptation approach was used to translate the scale into Chinese (Beaton et al., 2000; Guillemin et al., 1993), and we eventually established the SERAMS. The steps for the translation and adaptation are as follows:

#### 2.3.1 Step I and II: forward translation and synthesis

SERATS was translated into Chinese by two bilingual translators. One translator is a native English speaker, and the other is a native Chinese speaker. Our research team members and the two translators discussed and compared the two drafts from Step I. One common draft of the Chinese version of the SERATS was obtained.

#### 2.3.2 Step III: back translation

Two other Chinese-English bilingual translators back-translated the common Chinese draft SERATS into English, and two back-translated English versions of the SERATS were obtained.

#### 2.3.3 Step IV: cross-population and cross-contextual adaptation

Four translators (forward and back translators), two linguists, one epidemiologist, and our research team members formed an expert committee to review and adapt the scale.

Since SERATS was developed for clinical samples that received art therapy, and the subjects in this study consist of university students majoring in art and design, the scale required cross-population and cross-contextual adaptation. After reviewing the content of all scales in the above Chinese and English versions and discussing them, the experts modified items 2, 8, and 9 in the common draft of SERATS’s Chinese version mentioned above. They modified item 2 from “I am able to depict my feelings in art therapy” to “I am able to depict my feelings in art making”; and item 8 from “I apply the new behavior that I have been experimenting with in art therapy outside of the therapy setting” to “I apply the new behavior that I have been experimenting with in art making outside of the art making setting”; and item 9 from “I gain greater insight into my psyche through art therapy” to “I gain greater insight into my psyche through art making” to ensure that the adapted scale is appropriate for use by art students while retaining the core meaning of the scale. Finally, we obtained a new Chinese scale based on SERATS.

#### 2.3.4 Stage V: synthesis and finalization of the initial Chinese version

The expert committee analyzed and compared the SERATS, two forward-translated versions, one common translated draft, two back-translated versions, and the new Chinese scale adapted from SERATS. The expert committee considered that the new Chinese scale, adapted from SERATS, is equivalent to the original SERATS in terms of semantics, concepts, experience, and idiomatic expression, except for the differences in the applicable populations and context.

#### 2.3.5 Stage VI: pretesting and cognitive interviews

Thirty university students who met the research criteria participated in the pretesting and cognitive interviews to examine the comprehensibility of the new Chinese scale adapted from SERATS. All participants reported understanding each item in the questionnaire without ambiguity.

#### 2.3.6 Stage VII: finalization of the Chinese version of SERAMS

After discussion, the expert committee decided to accept the new Chinese scale, adapted from SERATS, and name it SERAMS.

### 2.4 Data collection

The participants completed the questionnaires online. The researcher created and edited the questionnaire in WJX software (powered by www.wjx.cn) on a computer, then generated a QR code for it. The researcher then shared the QR code in the WeChat group for undergraduate or graduate classes in each major, and participants scanned the code on their cell phones to access the questionnaire. The first page of the survey was an informed consent form; participants were required to provide their consent before proceeding to the questions, which they completed voluntarily. Each participant received 5 RMB via WeChat transfer upon completing the initial questionnaire. The questionnaire also included a question asking if participants were willing to take part in a retest in two weeks; those who volunteered provided their cell phone numbers as a unique identifier to allow for data matching. We randomly selected 121 people from those who agreed to participate, and two weeks after completing the initial questionnaire, they scanned a new QR code to complete the SERAMS online again. Upon completing the retest, these participants each received a notebook valued at 12 RMB, with six distinct designs available to choose from.

### 2.5 Statistical analysis

Raw data were exported from the WJX platform as Excel files and then imported into SPSS 23.0 for statistical analysis. The socio-demographic information of the subjects was summarized using mean and standard deviation, frequency, and percentage. Continuous variables were presented as mean ± standard deviation (SD), while counts and percentages were used for categorical variables. A *p*-value of less than 0.05 indicates a statistically significant difference. Additionally, AMOS 23.0 was utilized for the confirmatory factor analysis (CFA). A composite total score for the SERAMS was calculated for each participant by summing the responses across all nine items to facilitate the correlation analysis.

#### 2.5.1 Item analysis

Item analysis was performed using item-total correlation analysis to identify and eliminate items with a low correlation with the total SERAMS score. This process ensures that all items are internally consistent and measure the same construct. The removal criteria involve a Pearson correlation coefficient less than 0.4 or a non-significant difference test result (*p*>0.05) (Hair et al., 2009; Wu, 2010).

#### 2.5.2 Validity analysis

SERAMS’s content validity, construct validity, and criterion-related validity were evaluated.

Six senior faculty members from the Department of Art and Design at the Fine Art School assessed the SERAMS’s item-level content validity index (I-CVI) and scale-level content validity index (S-CVI). The I-CVI was calculated as the proportion of experts who assigned a rating of 3 (quite relevant) or 4 (very relevant) to each item. Items with an I-CVI of 0.78 or higher should be kept. If the S-CVI/Ave (the average scale-level content validity index) is 0.9 or higher, the scale-level content validity is acceptable (Lynn, 1986; Polit et al., 2007). Otherwise, unqualified items should be deleted or modified and re-evaluated until they meet the criteria.

We performed an exploratory factor analysis (EFA) to test the scale’s construct validity. If the Kaiser-Meyer-Olkin measure of sampling adequacy (KMO ≥ 0.70) and Bartlett’s Test of Sphericity was statistically significant (*p* < 0.05) (Bartlett, 1950, 1951), the scale was deemed suitable for factor analysis. Items with communalities below 0.2 should be excluded (Wu, 2010). If the Measure of Sampling Adequacy (MSA) for a particular item is greater than or equal to 0.5, the item can be included in the factor analysis. Otherwise, it should be removed (Spicer, 2005). We used principal component analysis (PCA) combined with the Varimax orthogonal rotation method—selected to achieve a simple structure and enhance interpretability—to analyze the data. The following criteria were applied to determine the number of factors: (1) Kaiser’s principle of eigenvalues greater than one (Kaiser, 1960). (2) Factors containing at least two items with loadings greater than 0.4 (Samuels, 2017). (3) Deletion of items with a cross-loading ratio greater than 75% (i.e., the secondary loading is more than 75% of the primary loading), following the criteria suggested by Samuels (2017). The scree test helped judge the PCA results.

In the confirmatory factor analysis (CFA), we used the following criteria to assess the model’s goodness. A model with a non-significant *p*-value for the χ2 test was considered a suitable model. The ratio of χ2 to degrees of freedom (CMIN/df) being less than 5, along with a goodness of fit index (GFI), Tucker-Lewis index (TLI), and comparative fit index (CFI) values greater than 0.9 indicate a robust model fit (Byrne, 1994). Additionally, a standardized root mean square residual (SRMR) less than 0.05 and a root mean square error of approximation (RMSEA) value of 0.08 or less further confirm that the model fits the data well (Hoyle, 1995; Wu, 2010).

We hypothesized a significant positive Pearson correlation coefficient between the SERAMS and the GSES; the criterion-related validity is considered acceptable if the coefficient value is≥0.4 (Kaplan and Saccuzzo, 2008).

#### 2.5.3 Reliability analysis

We evaluated the SERAMS’s internal consistency (Cronbach’s alpha), intraclass correlation coefficient (ICC), split-half reliability (Spearman-Brown coefficient), and test–retest reliability. Cronbach’s alpha≥0.70; its internal consistency reliability is acceptable. If removing an item would notably enhance the Cronbach’s alpha of the scale, this indicates that the item lacks homogeneity with the other items and will therefore be excluded from the scale (DeVellis, 2016). The split-half reliability is considered appropriate if the Spearman-Brown coefficient is≥0.70 (DeVellis, 2016). The intraclass correlation coefficient (ICC) between 0.6 and 0.74 is good (Cicchetti and Sparrow, 1981). A Pearson correlation coefficient *r* > 0.7 for test–retest reliability is acceptable (Vilagut, 2014).

#### 2.5.4 Ceiling effect and floor effect

We evaluated the ceiling and floor effects of the items. If over 15% of respondents reach the highest or lowest score on an item, it indicates a response bias in the data. There is a ceiling or floor effect on data (Terwee et al., 2007).

## 3 Results

Four hundred and nine participants were collected, from which 209 participants were randomly selected as subjects for the item, reliability, and validity analysis. The remaining 200 participants were included in the CFA. As participants submitted their responses only after completing the full questionnaire, and all questions were mandatory, no data was missing. Descriptive information for the characteristics of all the participants are provided in Table 1.

**Table 1:** General characteristics of the participants in Sample A and B.

**Characteristics**	**Sample A**	**Sample B**
*N*	209	200
Age(years)	20.32 ± 2.129	20.29 ± 1.924
Sex		
Male (%)	19.62%, (41/209)	19.50%, (39/200)
Female (%)	80.38%, (168/209)	80.50%, (161/200)
Sample size by grade		
Year 1 of bachelor	90	91
Year 2 of bachelor	71	52
Year 3 of Bachelor	18	16
Year 4 of bachelor	16	26
Year 1 of master	6	8
Year 2 of master	4	4
Year 3 of master	4	3
Sample size by major		
Environmental art design	27	39
Graphic design	26	32
Public art design	113	86
Fine art education	28	26
Painting	15	17
Total Score of GSES	24.98 ± 5.88	
Total Score of SERAMS	29.01 ± 5.11	29.65 ± 5.38

Data are means ± SD or percentages. N, the sample size; Sample A, Participants of item, reliability, and validity analysis; Sample B, Participants of CFA.

### 3.1 Item analysis

The correlation coefficient between SERAMS’s item scores and its total scale score ranged from 0.692 to 0.732, and the differences were statistically significant (*p* < 0.01). All nine items were retained (Table 2).

**Table 2:** Item analysis and EFA.

Items	*R*	MSA	IC	Factor loading
1	0.699^**^	0.924	0.489	0.699
2	0.718^**^	0.915	0.519	0.720
3	0.720^**^	0.903	0.526	0.725
4	0.731^**^	0.928	0.532	0.730
5	0.732^**^	0.932	0.538	0.733
6	0.692^**^	0.913	0.468	0.684
7	0.721^**^	0.937	0.519	0.720
8	0.697^**^	0.934	0.484	0.695
9	0.724^**^	0.906	0.527	0.726
Eigenvalue	4.601			
Variance explained (%)	51.124%			

**Correlation is significant at the 0.01 level; Extraction Method: Principal Component Analysis (Kaiser’s eigenvalue > 1); One component extracted. R, correlation coefficient of each item to the total score of SERATS; MSA, Measures of Sampling Adequacy; IC, Item’s Communalities.

### 3.2 Validity analysis

#### 3.2.1 Content validity

Six senior faculty members from the Department of Art and Design at the Fine Art School assessed SERAMS’s content validity. All nine items had an I-CVI of 1.0, higher than the acceptable threshold of 0.78 (Lynn, 1986). The scale-level content validity index (S-CVI) of SERAMS was calculated using the S-CVI/Ave method, which averages the I-CVI values for each item. This index was also 1.0, exceeding the cutoff value of 0.9 (Polit et al., 2007). The SERAMS exhibits good content validity (Lynn, 1986; Polit et al., 2007).

#### 3.2.2 Construct validity

**Figure 1:**
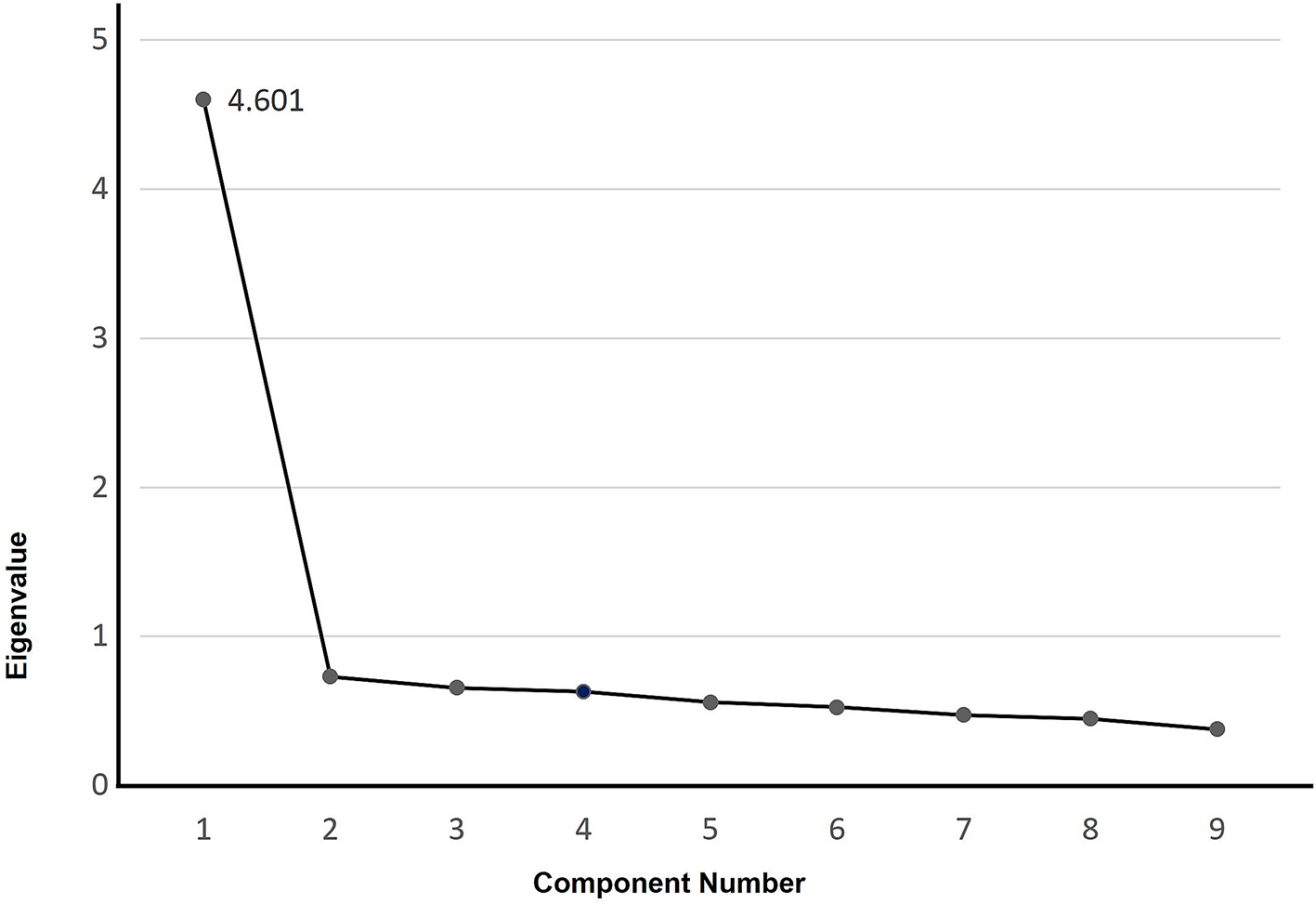
Scree plot. One factor has a Kaiser’s eigenvalue≥1; extraction method: principal component analysis.

Bartlett’s Test indicated that the SERAMS was suitable for factor analysis. The χ2 value was 698.815 (*p*<0.001) (Bartlett, 1950, 1951). The communalities of the items ranged from 0.468 to 0.538, all surpassing 0.2. Meanwhile, the MSA values for the items ranged from 0.903 to 0.937, all exceeding 0.5, indicating that all nine scale items were suitable for the EFA (Table 2). The Kaiser’s eigenvalue-over-one principle was used to extract a common factor, which explained 51.124% of the total variance. The initial eigenvalue was 4.601. The factor loadings of SERAMS items ranged from 0.684 to 0.733. Additionally, the scree plot indicated that extracting a common factor was appropriate (Figure 1). The results from both the Kaiser’s criterion and the scree plot analysis consistently supported a single-factor structure for the scale.

**Figure 2:**
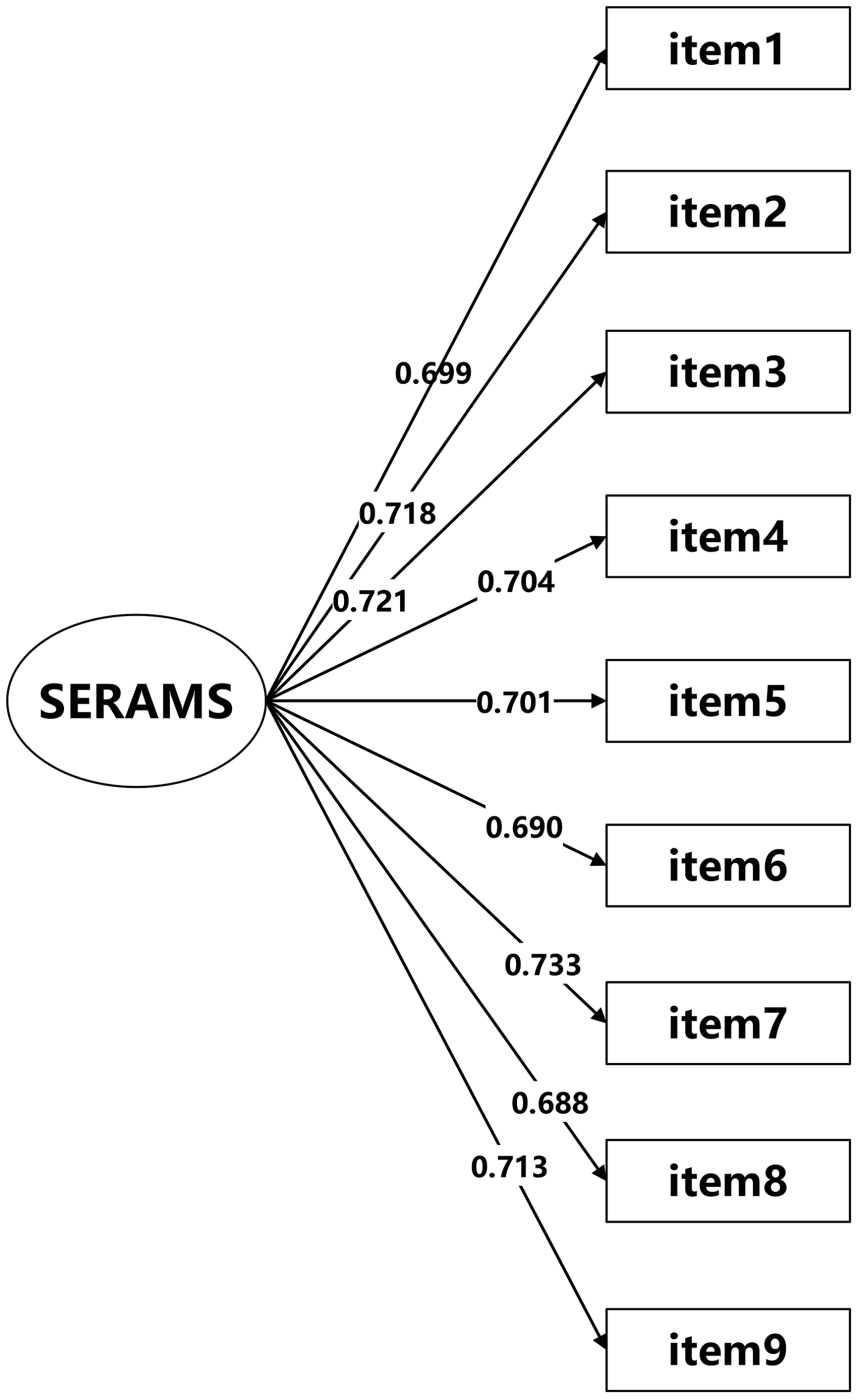
Path diagram for SERAMS.

Results of the CFA confirmed the structure of the SERAMS with a good model fit the data (χ2 = 33.837, df = 27, *p* = 0.171, CMIN/df = 1.253; RMSEA = 0.036, SRMR = 0.0304, TLI = 0.988, CFI = 0.991, GFI = 0.964). The results of the CFA are presented in Figure 2.

#### 3.2.3 Criterion-related validity

A positive correlation exists between the total score of SERAMS and GSES, with a Pearson correlation coefficient of *r* = 0.571 (*p* < 0.001). The criterion-related validity of the SERAMS is considered acceptable (Kaplan and Saccuzzo, 2008).

### 3.3 Reliability analysis

The Cronbach’s alpha coefficient of SERAMS was 0.880(p<0.001). Removing an item from SERAMS would not increase its Cronbach’s alpha value. All nine items in the SERAMS were retained. The Cronbach’s alpha of SERAMS are appropriate (DeVellis, 2016). SERAMS’s ICC was 0.880 (95% CI: 0.854–0.903), which is considered good according to Cicchetti’s criteria (Cicchetti and Sparrow, 1981). The Spearman-Brown coefficient was 0.875, indicating that SERATS’s split-half reliability was good (DeVellis, 2016). Two weeks later, we used SERAMS to re-test 121 subjects. The Pearson correlation coefficient for the SERAMS test–retest reliability was r = 0.889 (*p* < 0.001). The test–retest reliability of SERAMS was acceptable (Vilagut, 2014) (Table 3).

**Table 3:** Reliability of SERAMS.

Item number	Means ± SD	α
1	3.28 ± 0.779	0.869
2	3.32 ± 0.777	0.867
3	3.23 ± 0.745	0.866
4	3.26 ± 0.815	0.866
5	3.05 ± 0.804	0.865
6	3.09 ± 0.830	0.870
7	3.28 ± 0.804	0.867
8	3.25 ± 0.788	0.869
9	3.24 ± 0.803	0.866
Cronbach’s alpha = 0.880		
ICC: 0.880 (95% CI 0.854–0.903)		
Split-half reliability: Spearman-Brown coefficient = 0.875		
Test–retest reliability: *r* = 0.889		

α, Cronbach’s alpha without item.

### 3.4 Ceiling effect and floor effect

No participants achieved the lowest total score of 9. However, three participants scored the highest total score of 45, representing 0.14% of all individuals. Therefore, no subject response bias was observed in the current study (Terwee et al., 2007).

## 4 Discussion

Various reasons may have contributed to the problem of self-expression and emotion regulation in art making among Chinese art and design university students (Fielding and Chung, 1998; Hong, 2020; Zhao et al., 2020). Although this problem has long troubled art and design educators in China, the lack of suitable research tools has slowed progress in this field, hindering the advancement of art and design education.

SERATS is a good research tool for assessing self-expression and emotional regulation in art therapy. It has high internal consistency. Cronbach’s alpha of the nine items was 0.90–0.94, and the test–retest correlation was r = 0.96 (Haeyen et al., 2018; Haeyen and Noorthoorn, 2021). However, due to limitations in the applicable population and usage context, SERATS cannot be directly used to measure the self-expression and emotional regulation of Chinese art and design university students in their art making. After the cross-population and cross-contextual adaptation of SERATS, the resulting SERAMS has relatively good psychometric characteristics. Its Cronbach’s alpha coefficient was 0.880, and the test–retest correlation was *r* = 0.889. Notably, the final version of SERAMS had a similar structure to SERATS, consisting of nine items and one dimension. This is an encouraging outcome that demonstrates the structural stability and robust construct validity of the scale during cross-cultural adaptation.

Art therapy emphasizes using art as a tool for emotional catharsis and psychological healing (Gussak and Rosal, 2015), while art education focuses more on skill enhancement, originality of works, and professional growth (Runco, 2014). SERAMS retains the psychological core of SERATS while also serving the goals of art and design education. Therefore, SERAMS successfully bridges the two fields of art therapy and art and design education. Multiple studies have shown that appropriately adapted scales for clinical use can be used in the general population (Cooper et al., 1987; Krupp et al., 1989), and our research has once again confirmed this.

Correlation analysis of the SERATS with the Emotion Regulation Strategies for Artistic Creative Activities Scale (ERS-ACA) found that the SERATS was positively associated with ERS-ACA’s Approach Strategies factor (r = 0.69–0.81); Self-development Strategies factor (r = 0.53–0.62) (Haeyen and Noorthoorn, 2021). Since there is currently no Chinese version of the ERS-ACA, we were unable to conduct a correlation analysis between the SERAMS and the ERS-ACA. Evidence suggests that an individual’s sense of self-efficacy correlates with self-expression and emotional regulation in art and education (Alessandri et al., 2015; Kaimal and Ray, 2017; Khastari and Asgari, 2019; Luberto et al., 2014; Yusefi et al., 2024). Therefore, we used the GSES to assess the criterion-related validity of the SERAMS, which showed a positive correlation between the SERAMS and the GSES (*r* = 0.571, *p* < 0.001). General self-efficacy in art and design education reflects art students’ beliefs in their ability to do well in art making, and students with high general self-efficacy will express themselves more positively and regulate their emotions during art making, seeking to take control of the situation. However, the psychological processes involved in art making may be quite complex. In art making, students may need to confront and use the complex emotions they generate rather than simply believing they can control them, as general self-efficacy emphasizes. Therefore, general self-efficacy may be only one of many factors influencing artistic creation and cannot fully explain the psychological processes underlying it. This may be why there is a correlation between SERAMS and GSES, but not a strong one.

### 4.1 Educational implications

Given the positive correlation between the two, this suggests that improving students’ general self-efficacy may partially enhance their self-expression and emotion regulation in art making. For example, to encourage students to be more willing to express their inner world through art, teachers can design moderately challenging step-by-step tasks in art classes that students can successfully complete and experience a sense of achievement. For example, in art teaching, teachers and peers should provide specific, genuine, and positive feedback rather than vague phrases like “good job” to enhance students’ self-confidence.

Alternatively, show the creative process of successful artists or exemplary peers in teaching, discussing together the techniques and methods that underpin their success. Seeing similar people succeed would increase students’ confidence in their potential (Bandura, 1997). In addition, by improving students’ emotion regulation (Haeyen et al., 2018), teachers can instruct students to engage in relaxation exercises such as breathing meditation to manage their anxiety during the art making process.

### 4.2 Future research and directions

Due to constraints in research duration, the responsiveness of the SERAMS—specifically its ability to detect changes in students’ self-expression and emotion regulation over time or following interventions—was not assessed. Future research should adopt longitudinal or pre-post intervention designs to systematically evaluate this property.

Additionally, it is worth noting that emotion regulation and self-expression are two distinct psychological constructs. The items in SERATS emphasize both self-expression and emotion regulation, such as experiencing, becoming aware, and expressing feelings, regulating emotions/feelings by applying new behavior, and gaining insight (Haeyen et al., 2018). However, because the scale is unidimensional, it cannot clearly distinguish between these two dimensions. This is not an intentional design choice by the scale’s developers but rather a rational acceptance of the research findings based on statistical analysis, reflecting a scientific approach. Our SERAMS inherits this same limitation. In the future, it may be necessary to develop one standardized tool specifically for measuring self-expression in art making and another for measuring emotion regulation in art making to address this issue.

## 5 Strengths

To our knowledge, SERAMS is the first tool to assess the self-expression and emotional regulation of Chinese university students majoring in art and design during their art making process. Another strength is the systematic translation and cross-cultural adaptation process, which ensured the scale’s linguistic accuracy and contextual relevance for Chinese art students. The number of subjects in this study met the sample size requirements for a revised-scale study (Costello and Osborne, 2005; Wu, 2010). The objectives of the study and the inclusion and exclusion criteria for subjects were clearly defined. The SERAMS has relatively good reliability and validity, and its structure has been confirmed by confirmatory factor analysis (CFA). These factors represent the strengths of the study.

## 6 Limitations

Although the revisions to SERAMS have been successful, some shortcomings have emerged during the revision process and need to be addressed in the future. First, China has multiple subcultures across geographic regions, and its population has many different socioeconomic profiles. The subjects for this study were collected only from a university in an urban area, limiting the representativeness of SERAMS across different socioeconomic and regional contexts. Second, while a rigorous systematic translation process was followed, inherent cross-cultural nuances in artistic expression may still exist, representing an unavoidable challenge in cross-cultural semantic equivalence. Third, relying solely on self-reports from art students may yield biased results. Fourth, the pilot test was conducted only once due to time constraints, potentially missing the opportunity to refine the project. Fifth, the subjects for this study were drawn from five art majors: Environmental Art Design, Graphic Design, Public Art Design, Fine Art Education, and Painting. However, the adaptation of SERAMS overlooks potential differences among the aforementioned art subdisciplines, potentially leading to the loss of information unique to each subdiscipline. The above issues remain to be gradually improved in future research efforts.

## 7 Conclusion

We revised the SERAMS using Chinese university students majoring in art and design as the research subjects. The scale has shown relatively good psychometric properties, with one dimension and nine items. It can be used to assess the self-expression and emotion regulation of Chinese university students majoring in art and design during their art making process.

## Data Availability

The original contributions presented in the study are included in the article/supplementary material, further inquiries can be directed to the corresponding author/s.
